# Pulp Stone Formation Following Fixed Orthodontic Treatment: A Panoramic Radiographic Comparison of Extraction and Non‐Extraction Approaches

**DOI:** 10.1002/cre2.70181

**Published:** 2025-07-24

**Authors:** Kosar Gholinezhad, Hakimeh Ghorbani, Seyedali Seyedmajidi, Sedigheh Sheikhzadeh, Manouchehr Rahmati Kamel

**Affiliations:** ^1^ Student Research Committee Babol University of Medical Sciences Babol Iran; ^2^ Oral Health Research Center, Health Research Institute Babol University of Medical Sciences Babol Iran; ^3^ Dental Materials Research Center, Health Research Institute Babol University of Medical Sciences Babol Iran

**Keywords:** orthodontic extraction, panoramic radiography, pulp calcification, pulp stone formation

## Abstract

**Objective:**

The impact of orthodontic forces on pulp stone formation has been the focus of several studies. Given that orthodontic extractions typically involve the application of greater forces to the teeth, the aim of this study was to compare the extent of pulp stone formation in the molar teeth of patients undergoing orthodontic treatment with and without extractions.

**Material and Methods:**

In this retrospective observational study, panoramic radiographs of 80 orthodontic patients taken between 2014 and 2020 (equally divided into extraction and non‐extraction groups) who had a full set of permanent molars were analyzed before and after orthodontic treatment to assess the formation of pulp stones in the pulp chambers of the molar teeth (640 molars). Data were analyzed using the Chi‐square and McNemar tests with a significance level set at *p* < 0.05, using SPSS software.

**Results:**

The frequency of pulp stone formation significantly increased in both the extraction and non‐extraction groups following fixed orthodontic treatment (*p* < 0.001 and *p* = 0.02, respectively). However, no statistically significant difference was observed in the extent of pulp stone formation between the two groups (*p* = 0.09). The frequency of patients exhibiting pulp stone formation did not differ significantly by gender in either the extraction or non‐extraction treatment groups (*p* = 0.392 and *p* = 0.451, respectively). In the extraction group, the prevalence of pulp stones was significantly higher in the first molar compared to the second molar (*p* = 0.001). In contrast, no significant difference was found between the first and second molars in the non‐extraction group (*p* = 0.108). Additionally, no correlation was found between the frequency of pulp stone formation and jaw type (maxilla or mandible) in either group (*p* > 0.05).

**Conclusions:**

Fixed orthodontic treatment is associated with increased pulp stone formation, regardless of whether extractions are performed. These findings may help clinicians in the early identification and monitoring of at‐risk teeth.

## 1 Introduction

Pulp stones, also known as dental pulp calcifications, are calcified structures that can be found within the pulp of both healthy and compromised teeth, as well as unerupted teeth. These calcifications may be either free‐floating or attached to the pulp tissue, with sizes ranging from microscopic particles to large masses that may obstruct the pulp chamber (Chalikkandy et al. 2023; Talla et al. 2014). Radiographically, pulp stones are typically observed as single or multiple radiopacities within the pulp chamber or root canal system (Ertas et al. 2017; Patil and Sinha 2013). Despite extensive research, the precise etiology of pulp stone formation remains uncertain, and there is currently no definitive evidence linking them to specific systemic or pulp‐related disorders. However, factors such as advancing age, genetics, systemic diseases, pulp degeneration, interactions between pulp tissue and the surrounding epithelium, impaired blood circulation within the pulp, bacterial infections, and orthodontic tooth movements have all been proposed as potential contributing factors (Andreasen et al. 2018; Berès et al. 2016; Jain et al. 2014; Kaswan et al. 2014; Khan et al. 2024; Maikhuri et al. 2023; Nayak et al. 2010; Patil and Sinha 2013).

Although pulp stones are generally asymptomatic, they can pose clinical challenges. In certain cases, large pulp stones may obstruct the canal orifice, alter the internal anatomy of the tooth, complicate root canal procedures by hindering file navigation, and potentially lead to treatment failure (Gulsahi et al. 2009; Palatyńska‐Ulatowska et al. 2021). Additionally, the presence of pulp stones can complicate access to root canals, occasionally necessitating tooth extraction (Aleksova 2015; Gambarini et al. 2020).

The relationship between orthodontic treatment and pulp stone formation has been explored in various studies. Orthodontic forces are known to induce complex tissue reactions that affect both the pulp and periodontal structures, with potential alterations in blood flow and vascular pressure that may contribute to calcification processes within the pulp (Farshidfar et al. 2022; Maikhuri et al. 2023).

The formation of pulp stones following orthodontic treatment has been documented, with some studies reporting increased prevalence posttreatment. For instance, Afsari et al. observed a significant increase in pulp stones after treatment (Afsari et al. 2021), while Jena et al. (2018) found a 4% increase in prevalence in premolar and molar teeth using panoramic radiographs. Similarly, Ertas et al. (2017) noted a rise from 3% to 5.2% in pulp stone occurrence, particularly in maxillary molars, and Maikhuri et al. (2023) reported a significant increase in the incidence of pulp stones at the conclusion of orthodontic treatment, with the first molars of the maxilla showing the highest frequency of formation.

Given the evidence suggesting that orthodontic treatment may promote pulp stone formation, the impact of treatment modality—particularly extraction versus non‐extraction approaches—remains unclear. Since extraction cases typically involve greater tooth movement and force application, the aim of this study was to compare the extent of pulp stone formation in molar teeth following fixed orthodontic treatments in extraction and non‐extraction cases using panoramic radiographs.

## 2 Materials and Methods

### 2.1 Study Design and Sample

This retrospective observational study was conducted on panoramic radiographs of orthodontic patients who were referred to the Department of Orthodontics at the School of Dentistry between 2014 and 2020 for fixed orthodontic treatment. The minimum required sample size was calculated based on data from a previous study by Korkmaz et al. (2019), which reported an increase in the prevalence of pulp stones from 7.1% before orthodontic treatment to 31.7% after treatment. Using these proportions, we applied the following formula for comparing two independent proportions:

$$n=\frac{{\left(Z_{1-\frac{\alpha }{2}}+Z_{1-\beta }\right)}^{2}[P1\left(1-P1\right)+P2\left(1-P2\right)]}{{\left(P1-P2\right)}^{2}}$$

where

- α = 0.05 (95% confidence level), so Z_1−α/2_ = 1.96
- β = 0.2 β = 0.2 (80% power), so Z_1−β_ = 0.84
- P1 = 7.1% and P2 = 31.7% (Korkmaz et al. 2019).

Substituting these values into the formula:

$$n=\frac{{\left(1.96+1.28\right)}^{2}[\left(0.071\times 0.929\right)+\left(0.317\times 0.683\right)]}{{\left(0.071-0.317\right)}^{2}}=36.6$$

Thus, a minimum of 37 subjects per group was required. To enhance statistical power and accommodate potential exclusions, 40 panoramic radiographs were selected for each group—extraction and non‐extraction—resulting in a total sample size of 80.

**Extraction Group:** Patients who underwent orthodontic treatment involving the extraction of at least two premolars as part of space‐gaining mechanics to address crowding or protrusion, typically to allow for retraction of anterior teeth or correction of severe malocclusions (Proffit et al. 2019).

**Non‐Extraction Group:** Patients who completed orthodontic treatment without any extractions, often managed by dental arch expansion, interproximal reduction, or distalization strategies, usually suitable for mild to moderate crowding (Proffit et al. 2019).

### 2.2 Inclusion Criteria

- Patients with a full set of permanent molars (first and second molars).
- Availability of pre‐ and post‐treatment panoramic radiographs.
- No history of systemic diseases, dental trauma, or endodontic treatment.

### 2.3 Exclusion Criteria

- Molars with root canal treatment or congenital absence.
- Poor radiographic quality or incomplete records.
- Patients with a history of systemic diseases.
- Extensive dental caries or restorations.

### 2.4 Sample Screening and Tooth Assessment

Pre‐ and posttreatment panoramic radiographs were retrieved from the patients' records. A total of 640 molars (8 molars per patient) were assessed for the presence of pulp stones. All radiographs were examined by a radiologist who was blinded to both the timing (pre‐ or post‐treatment) and the treatment group (extraction or non‐extraction).

**Figure 1:**
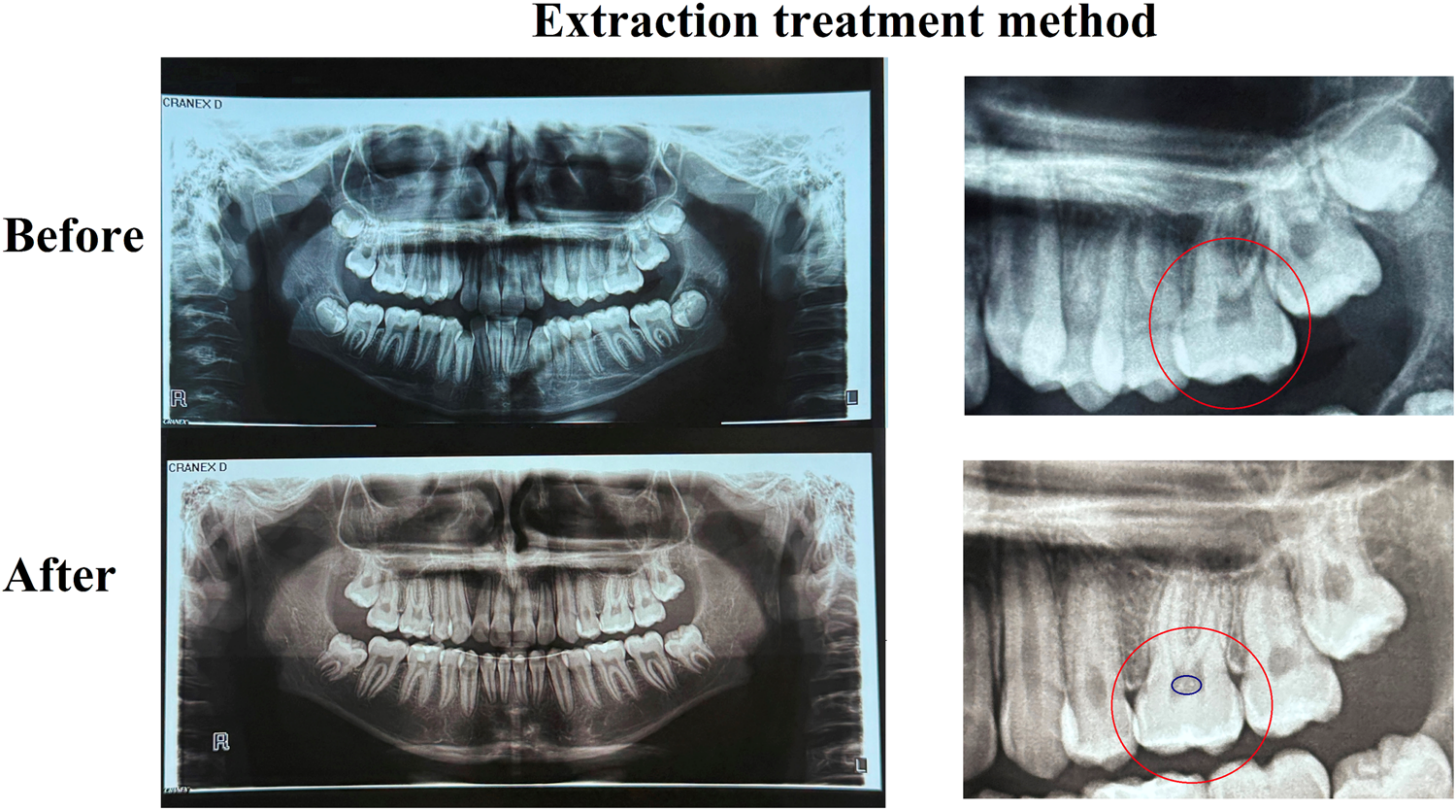
Pre‐ and post‐treatment radiographs of a patient who underwent fixed orthodontic treatment with the extraction method. The red circle highlights the tooth that developed a pulp stone, while the blue circle indicates the location of the pulp stone(s).

**Figure 2:**
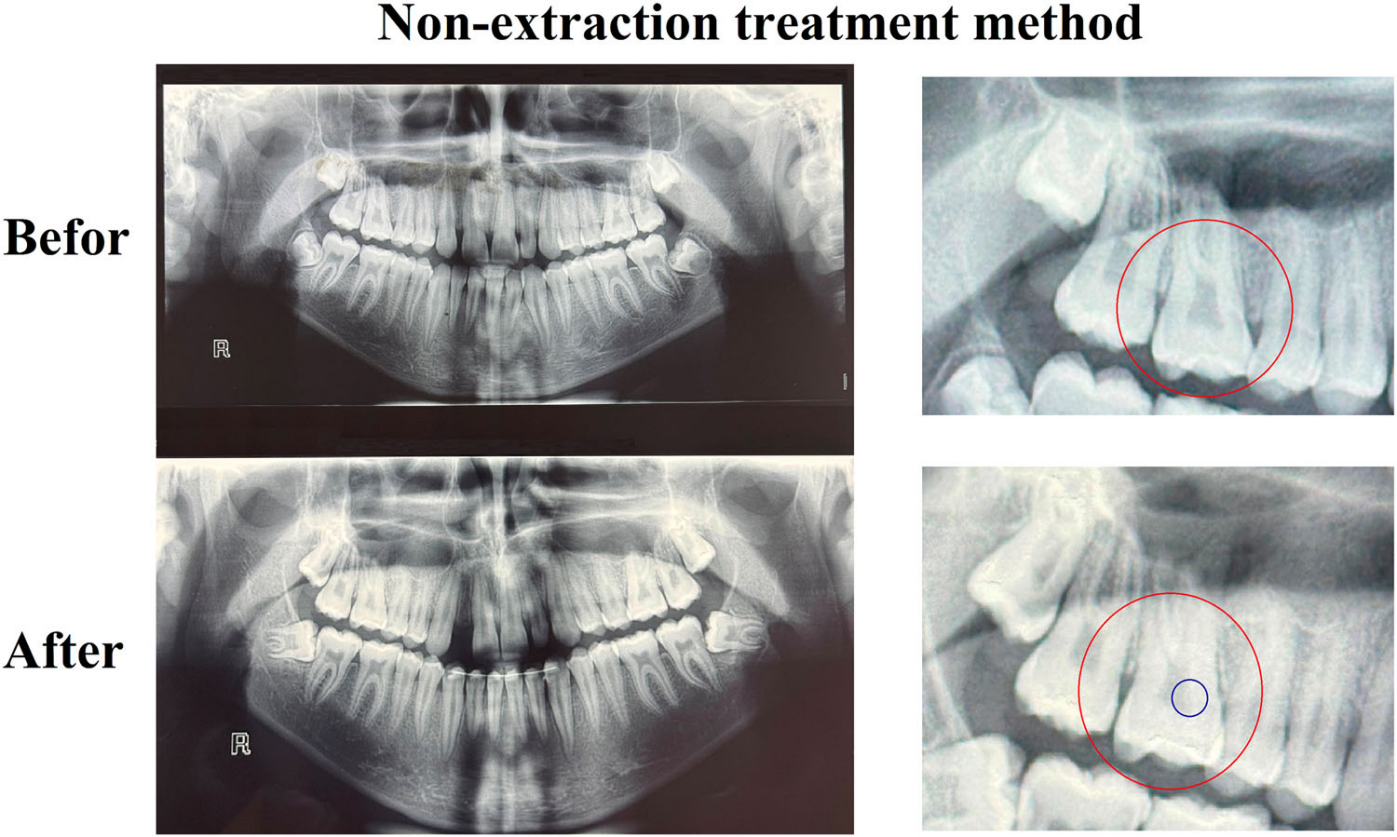
Pre‐ and post‐treatment radiographs of a patient who underwent fixed orthodontic treatment with non‐extraction method. The red circle highlights the tooth that developed a pulp stone, while the blue circle indicates the location of the pulp stone(s).

Each image was evaluated using a magnifying lens (2× to 5× magnification) at a viewing distance of approximately 30 cm. A pulp stone was definitively diagnosed only when the observer was certain, after two independent examinations, that a radiopaque mass within the pulp chamber was indeed a pulp stone (Figures 1 and 2).

### 2.5 Statistical Analysis

The collected data were analyzed using SPSS software (version 22). To evaluate the formation of pulp stones after treatment compared to before treatment within each group, the McNemar test was applied. To compare the extent of pulp stone formation based on treatment type (extraction vs. non‐extraction), tooth number (first vs. second molars), and jaw (maxilla vs. mandible), the Chi‐square test was used. A *p*‐value of < 0.05 was considered statistically significant.

## 3 Results

The study included 80 patients, comprising 19 males (23.75%) and 61 females (76.25%), with a mean age of 14.61 ± 3.42 years. The mean ages were comparable between the extraction (14.65 ± 3.92 years) and non‐extraction (14.58 ± 2.89 years) groups. The male‐to‐female ratio was 1:4.7 in the extraction group and 1:2.3 in the non‐extraction group. No statistically significant differences were observed in age (*p* = 0.923) and gender distribution (*p* = 0.293) between the two treatment groups, indicating a balanced demographic characteristic across both groups.

As shown in Table 1, among the 40 patients in the extraction group, 8 had pulp stones before treatment, and this number increased to 23 following treatment (*p* < 0.001). In the non‐extraction group, 11 patients exhibited pulp stones before treatment, which increased to 22 after treatment (*p* = 0.001).

**Table 1:** Changes in the frequency of patients with pulp stones before and after orthodontic treatment, categorized by treatment type.

Posttreatment	Extraction			Non‐extraction		
Pretreatment	Absent	Present	Total	Absent	Present	Total
**Absent**	17	15	32	18	11	29
**Present**	0	8	8	0	11	11
**Total**	17	23	40	18	22	40

**Figure 3:**
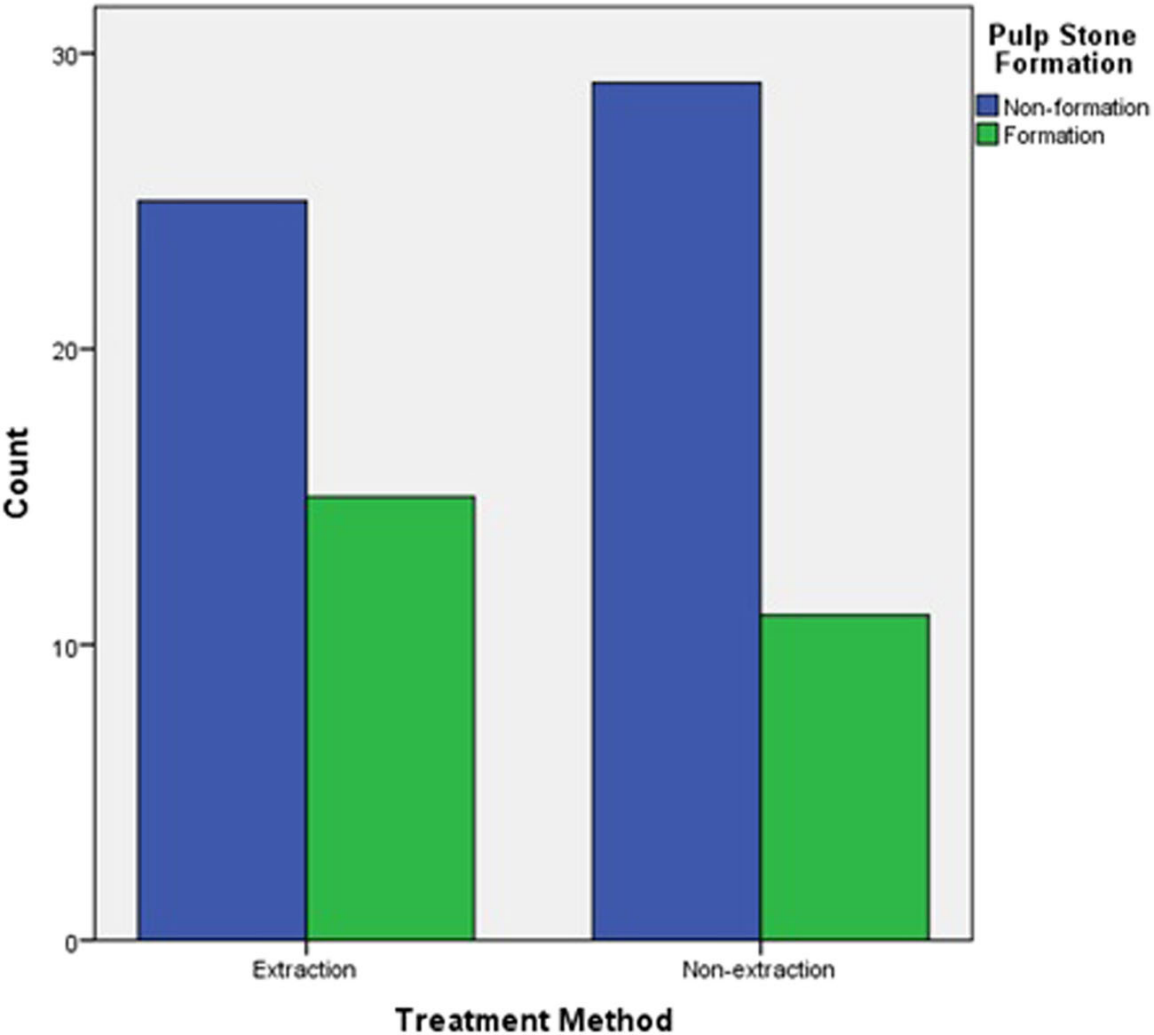
Frequency of patients exhibiting pulp stone formation after orthodontic treatment, categorized by treatment method.

The increase in the frequency of patients with pulp stones between the extraction and non‐extraction treatment groups was not statistically significant (*p* = 0.34) (Figure 3).

As shown in Table 2, the frequency of patients exhibiting pulp stone formation did not differ significantly by gender in either the extraction or non‐extraction treatment groups (*p* = 0.392 and *p* = 0.451, respectively).

**Table 2:** Frequency of patients exhibiting pulp stone formation (PSF) after orthodontic treatment in each group, categorized by gender.

Treatment method	Extraction			Non‐extraction		
Gender	Non‐PSF	PSF	*p* value*	Non‐PSF	PSF	*p* value*
Male	3 12%	4 26.7%	0.392	10 34.5%	2 18.2%	0.451
Female	22 88%	11 73.3%		19 65.5%	9 81.8%	
Total	25 100%	15 100%		29 100%	11 100%	

* Fisher's exact test.

The number of pulp stones in the extraction group increased significantly from 13 to 44 (*p* < 0.001). The distribution of the 31 newly formed pulp stones, based on jaw type and tooth number, is detailed in Table 3.

**Table 3:** Distribution of pulp stone formation by jaw and tooth type in the extraction group.

Dentin Jaw	First molar	Second molar	Total	*p* value*
Maxilla	**16**	**3**	19	0.209
Mandible	**9**	**3**	12
Total	25	6	31	
*p* value*	0.001		

* Chi‐square.

In the extraction group, pulp stone formation was significantly higher in the first molars compared to the second molars (*p* < 0.001), while no statistically significant difference was observed in pulp stone formation between the upper and lower jaws (*p* = 0.209).

In the non‐extraction group, the number of pulp stones also significantly increased from 24 to 43 following orthodontic treatment (*p* = 0.02). The distribution of the 19 additional pulp stones by jaw and tooth type is presented in Table 4.

**Table 4:** Distribution of pulp stone formation by jaw and tooth type in the non‐extraction group.

Dentin Jaw	First molar	Second molar	Total	*p* value*
Maxilla	**9**	**3**	12	0.251
Mandible	**4**	**3**	7
Total	13	6	19	
*p* value*	0.108			

* Chi‐square.

In the non‐extraction group, no statistically significant differences were observed in pulp stone formation between the first and second molars, nor between the upper and lower jaws (*p* = 0.251 and *p* = 0.108, respectively).

Additionally, the frequency of pulp stone formation did not differ significantly between patients treated with extraction versus non‐extraction orthodontics (*p* = 0.09).

## 4 Discussion

The number of patients with pulp stones significantly increased following fixed orthodontic treatment in both the extraction and non‐extraction groups. However, the difference in the extent of this increase between the two groups was not statistically significant. A similar pattern was observed in the total number of pulp stones. In both groups, pulp stone formation was more frequent in the first molars compared to the second molars, although this difference was statistically significant only in the extraction group. Additionally, while the frequency of pulp stone formation was higher in the maxilla than in the mandible in both groups, this difference did not reach statistical significance in either group. Moreover, the investigation of pulp stone formation based on gender within each group showed no statistically significant difference.

Both histological and radiographic evaluations can be used to detect pulp stones (Javed et al. 2015). While histological assessment is more reliable than radiographic evaluation, it is an invasive method. Pulp stones must reach a certain size (greater than 200 micrometers) to be detectable on radiographs (Gulsahi et al. 2009; Ranjitkar et al. 2002). One disadvantage of using radiography is that the interpretation may vary among different observers or even within the same observer over time (Deva et al. 2006). In the present study, a definitive diagnosis was made when the observer was certain, after two independent examinations, that a radiopaque object was a pulp stone.

Cone‐beam computed tomography (CBCT) provides detailed three‐dimensional anatomical views and has been utilized in recent studies to assess pulp stones (da Silva et al. 2017; Hsieh et al. 2018). However, due to its relatively high radiation dose, CBCT is not recommended for routine use in all orthodontic patients. Therefore, in the present study, pulp stones were evaluated using panoramic radiographs taken before and after orthodontic treatment.

Caviedes‐Bucheli et al. (2011) and Lazzaretti et al. (2014) found that orthodontic forces induce vascular changes in the pulp and promote an increase in pulp calcification. According to these studies, the mechanical forces applied during orthodontic treatment stimulate the release of various cytokines, including calcitonin gene‐related peptide and alkaline phosphatase, which play a role in the pulp's mineralization processes.

Studies have demonstrated that pulp stone formation is associated with various systemic diseases and syndromes, including type 1 diabetes, atherosclerosis, osteitis deformans, cardiovascular diseases, kidney diseases, dentin dysplasia, Marfan syndrome, Van der Woude syndrome, Saethre–Chotzen syndrome, Elfin facies syndrome, and familial expansile osteolysis (Bauss et al. 2008; Edds et al. 2005; Goga et al. 2008). Consequently, patients with these conditions were excluded from the present study. Additionally, due to the potential association between dental caries, extensive restorations, and pulp stone formation, individuals with these conditions were also excluded from the study (Jain et al. 2014; Patil and Sinha 2013).

The findings of this study are in agreement with those of Ertas et al. Afsari et al. Korkmaz et al. and Jena et al. who reported that fixed orthodontic treatment without extractions can lead to an increase in the number of pulp stones (Afsari et al. 2021; Ertas et al. 2017; Jena et al. 2018; Korkmaz et al. 2019). However, the study by Sarang (2018) did not observe any significant difference in pulp stone formation before and after orthodontic treatment.

In the present study, patients who underwent fixed orthodontic treatment with extraction were also evaluated, and both the frequency of patients with pulp stones and the total number of pulp stones in this group significantly increased. Contrary to the hypothesis of the study, which anticipated a greater formation of pulp stones in the extraction group due to increased tooth movement and higher applied forces, no statistically significant difference was observed in the increase in either the number of patients with pulp stones or the total number of pulp stones between the two groups. One possible explanation for this could be that the orthodontic forces applied in both treatment approaches were maintained within the biological limits of the periodontal ligament. When forces remain within this physiological range, they may not elicit markedly different pulpal stress responses, regardless of whether the treatment involves extractions. This suggests that it is not merely the extraction status, but rather the magnitude and control of applied forces that may influence the occurrence of pulp stone formation. This finding is consistent with the study by Babanouri et al. (2023).

Regarding pulp stone formation in the first and second molars, no significant difference was observed between the two in the non‐extraction group. However, in the extraction group, pulp stone formation was significantly higher in the first molars compared to the second molars after treatment, which is consistent with the findings of Ertas et al. (2017) and Jena et al. (2018). In contrast, Afsari et al. reported a greater increase in the number of pulp stones in the second molars following orthodontic treatment (Afsari et al. 2021). The reason for this discrepancy remains unclear and could be related to the differences in methodology and sample size of studies. Furthermore, it is uncertain whether the forces applied during treatment were different between the first and second molars. This issue warrants further investigation in future studies.

Several studies have shown that pulp stones are more prevalent in molars compared to other teeth (Babanouri et al. 2023; da Silva et al. 2017; Movahhedian et al. 2018; Patil et al. 2018). This may be attributed to the larger size of molars and their increased blood supply, which could facilitate a higher likelihood of calcification and pulp stone formation.

In the present study, no statistically significant difference was observed in pulp stone formation between the maxilla and mandible, regardless of whether the patients underwent extraction or non‐extraction treatment. This contrasts with the findings of Afsari et al. (2021), who reported a significantly greater increase in pulp stones in the mandible compared to the maxilla, while Ertas et al. (2017) found a higher frequency of pulp stones in the maxilla.

One of the limitations of the present study is the absence of skeletal malocclusion parameters in our analysis. This was primarily due to the constraints of the available panoramic radiographic data, which do not provide adequate information for assessing skeletal classifications. Future research incorporating cephalometric analysis could provide a more comprehensive understanding of the role that skeletal discrepancies may play in pulp stone development. Given the variability in results, it is recommended that future studies include follow‐up evaluations that assess both the extent of tooth movement and the forces applied during treatment, to establish a more accurate and definitive correlation between these factors and pulp stone formation following orthodontic treatment.

## 5 Clinical Implications

The results of this study underscore the importance of applying controlled, biologically appropriate forces during orthodontic treatment, regardless of whether extractions are involved. Clinicians should be aware of the potential for pulp stone formation as a post‐treatment outcome and consider incorporating pre‐ and post‐treatment radiographic evaluations when planning endodontic procedures. Understanding that pulp stone formation may not significantly differ between extraction and non‐extraction approaches can aid in more accurate risk assessment and treatment planning, particularly for patients with predisposing factors or complex restorative needs.

## 6 Conclusion

Fixed orthodontic treatment is associated with increased pulp stone formation, regardless of whether extractions are performed. These findings may help clinicians in the early identification and monitoring of at‐risk teeth.

## Author Contributions

Kosar Gholinezhad contributed to the acquisition of data, drafting of the article, and final approval of the manuscript. Hakimeh Ghorbani contributed to the acquisition of data. Sedigheh Sheikhzadeh contributed to the conception and design of the study. Seyedali Seyedmajidi contributed to the analysis of data, critical revision, and final approval of the manuscript. Manouchehr Rahmati Kamel contributed to the conception and design of the study, critical revision, and final approval of the manuscript.

## Ethics Statement

This study was approved by the Ethics Committee of Babol University of Medical Sciences (IR. MUBABOL.REC.1400.130).

## Conflicts of Interest

The authors declare no conflicts of interest.

## Data Availability

The data that support the findings of this study are available from the corresponding author upon reasonable request.
